# Microplastics in Foods Intended for Health Purposes: From Dietary Supplements to Clinical Nutrition Products

**DOI:** 10.3390/toxics14060514

**Published:** 2026-06-12

**Authors:** Kornelia Kadac-Czapska, Justyna Ośko, Katarzyna Jażdżewska, Małgorzata Grembecka

**Affiliations:** Department of Bromatology, Faculty of Pharmacy, Medical University of Gdańsk, 80-416 Gdansk, Poland; kornelia.kadac-czapska@gumed.edu.pl (K.K.-C.); justyna.osko@gumed.edu.pl (J.O.); katarzyna.jazdzewska@gumed.edu.pl (K.J.)

**Keywords:** microplastics, dietary supplements, herbal teas, honey, infant formula, enteral nutrition, parenteral nutrition, exposure assessment

## Abstract

Microplastics (MPs) are pervasive contaminants that have been detected throughout the food chain. Their presence raises concerns in foods intended for health-related purposes, as these products are often consumed by vulnerable populations such as infants, older adults, and patients requiring clinical nutrition support. These groups may be more susceptible to contaminant exposure and may rely heavily on specialized foods. Therefore, understanding the occurrence and potential risks of MPs in such products is important. This review summarizes the current state of knowledge regarding the presence, sources, and health implications of plastic particles in several categories of health-oriented foods, including dietary supplements, medicinal herbs, plant-based beverages, honey, infant formulas, and clinical nutrition products, including enteral and parenteral formulations. Microplastics have been reported across these matrices. Fibers and fragments dominate, and common polymers include polyamide, polyethylene, polypropylene, and poly(ethylene terephthalate). These particles can originate from polluted water, soil, and air, as well as from production processes, packaging wear, and clinical delivery systems. Current evidence suggests that improving methodological consistency and expanding targeted toxicological research relevant to vulnerable populations will be crucial for strengthening risk assessment.

## 1. Introduction

**Figure 1:**
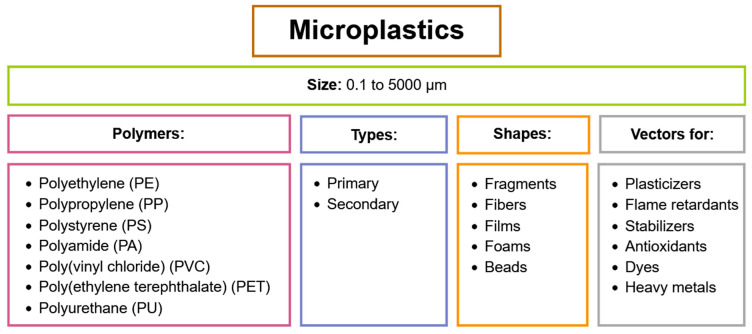
Physicochemical properties of MPs. Created by the authors using Canva Pro, https://www.canva.com/ (accessed on 11 May 2026).

Microplastics (MPs) are defined as plastic particles with a size range of 0.1 to 5000 µm [1,2]. They are divided into two categories: primary and secondary [3]. Primary MPs are particles manufactured at microscopic sizes or intentionally added to commercial products, such as personal care items. However, primary MPs generally account for a smaller fraction of environmental MPs than secondary MPs, and regulatory measures have increasingly targeted their intentional use. For example, the European Union has restricted the placing on the market of intentionally added synthetic polymer microparticles, including their use in certain personal care products, with transition periods depending on product type [4]. Secondary MPs are formed by the fragmentation of larger plastic materials under the influence of UV radiation, temperature, and mechanical abrasion [5]. These particles have become pervasive contaminants in food and the environment [6]. Microplastics vary widely in terms of their shape, color, size, and chemical composition (Figure 1) [7,8].

It has been hypothesized that small, fiber-shaped particles may have greater biological effects than regularly shaped particles, such as spheres [9]. Irregular particles may cause greater mechanical damage to tissues and cells under certain exposure conditions [10]. Additionally, MPs can function as vectors for various chemical substances (e.g., dyes, plasticizers, heavy metals) and microorganisms [11]. To date, MPs have been detected in various foods, including water, beverages, fruit, vegetables, dairy products, meat, fish, and edible oils [12,13,14,15,16]. MPs can enter the human body mainly through ingestion and inhalation, with dermal contact considered a possible but less established route. The primary route of exposure to MPs is ingestion [17].

Foods intended for health purposes include products used to supplement the diet or support specific nutritional needs, such as dietary supplements and clinical nutrition products. These products are available in a variety of forms and packaging types [18]. Contamination with MPs may occur during manufacturing, packaging, and transportation [19]. Their use by susceptible populations, such as infants and individuals with compromised health, is of particular concern because these groups may have developing or impaired physiological functions. Depending on their intended use, these products may supplement the diet with essential nutrients, such as vitamins and mineral components, or provide partial or complete nutritional support [20]. Infant formula and clinical nutrition products can serve as primary sources of nutrition and may therefore represent important sources of dietary exposure to MPs. Furthermore, dietary exposure to MPs may increase when plastic packaging, such as bottles, or other plastic components, such as tubing, are used [21].

The analysis of MPs in foods intended for health purposes is a relatively new and still underexplored area of research. Currently, there are no legal requirements for monitoring MPs in such foods [22,23]. Furthermore, few studies have analyzed the presence of MPs in these products [24,25,26,27]. Additionally, evaluating human exposure to MPs from food poses considerable challenges. This is due to the lack of standardized and universally validated methods for detecting and quantifying MPs [28].

The objective of this review is to summarize recent research findings on the occurrence of MPs in food products intended for health purposes. In addition, the review outlines potential pathways by which MPs may contaminate these products and discusses possible exposure scenarios based on selected examples, including dietary supplements, herbs, plant-based beverages, honey, infant formula, and clinical nutrition products.

## 2. Materials and Methods

This narrative review was based on publications indexed in PubMed, ScienceDirect, and Scopus. The search primarily covered studies published between 2021 and 2026; however, earlier papers were also considered when they provided substantial background or key evidence relevant to the topic. The search strategy included the following terms intitles, keywords, and abstracts: “microplastics” AND (“contamination sources” OR “water” OR “soil” OR “processing” OR “packaging” OR “bottle” OR “infusion bag” OR “dietary supplement” OR “herb” OR “plant” OR “tea” OR “honey” OR “infant formula” OR “enteral nutrition formula” OR “parenteral nutrition” OR “exposure assessment”).

Both original research articles and relevant review papers addressing exposure to MPs were eligible for inclusion. Review articles were used primarily to provide background information, identify broader research gaps, and support the interpretation of the evidence. Original studies were prioritized when reporting data on the occurrence of MPs and related exposure. The inclusion criteria comprised English-language publications, including studies focused on foods intended for health-related purposes. The exclusion criteria were non-English publications and sources for which full-text access was unavailable, including abstract-only records. After duplicate records identified across databases were removed, 129 scientific publications were included in the final analysis.

## 3. Contamination Sources

The production of health-oriented foods involves advanced technologies and strict hygiene standards. As a result, the risk of plastic contamination may be lower than in many conventional food products, although it cannot be completely eliminated [24].

**Figure 2:**
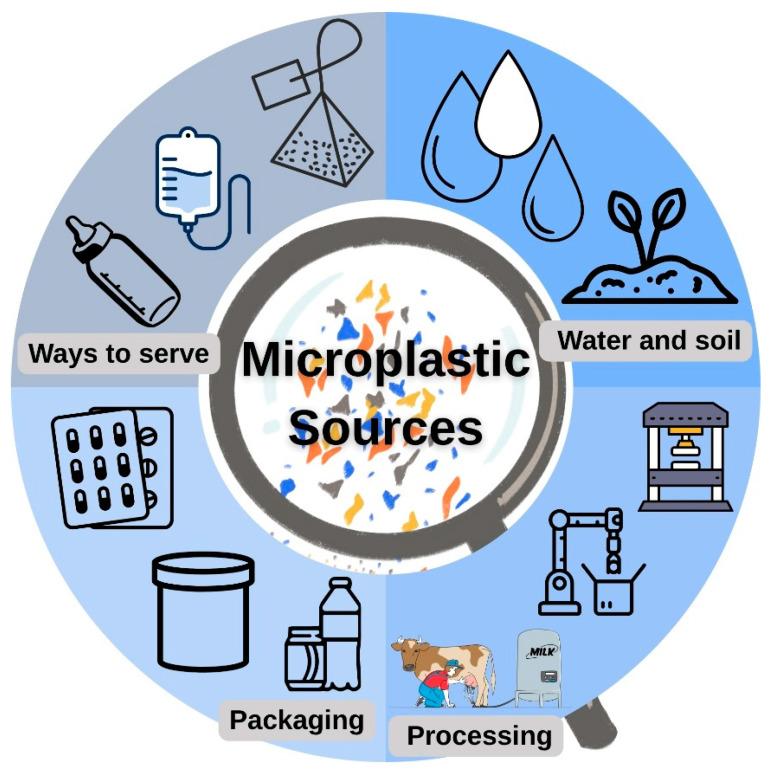
Potential sources of MPs in foods intended for health-related purposes. Created by the authors using Canva Pro, https://www.canva.com/ (accessed on 11 May 2026).

The sources of MPs in health-oriented foods are diverse. They may include contaminated raw materials, particularly those exposed to polluted soil or water, as well as processing and packaging stages [29]. Product use and administration practices may also contribute to contamination (Figure 2). Therefore, caution is required at each stage to minimize the risk of cross-contamination with MPs [24].

### 3.1. Water and Soil

Microplastics contaminating plant-based raw materials originate mainly from water and soil [27]. These particles can enter aquatic environments through multiple pathways, largely associated with human activity. Therefore, water used as a raw material or during production and cleaning may represent an important source of plastic contamination. The type of water source, such as surface water or groundwater, is also important [27]. The literature reports concentrations of MPs in surface waters reaching up to 809,000 particles/m^3^ [30]. Such contamination is linked to intensive human activity, including agricultural runoff, industrial emissions, wastewater discharge, and the fragmentation of plastic waste. Atmospheric transport may also play an important role. Microplastics can be carried by wind and enter water bodies along with precipitation.

For a long time, groundwater was considered less vulnerable to this type of pollution. However, the presence of plastic particles in these resources has also been confirmed. Contamination may occur when particles are washed out of soil and migrate deeper layers of the soil profile. In Europe, concentrations of 0.034–0.06 MPs/L have been reported, whereas higher values have often been observed in Asia, Australia, and South America [31,32,33,34]. Due to the long residence time of groundwater, MPs may persist in this environment for many years. This poses a long-term risk to groundwater quality. These pathways are particularly relevant for plant-derived ingredients and selected natural products used in health-oriented foods, including medicinal herbs, herbal teas, spirulina-based supplements, plant-based beverages, botanical ingredients used in dietary supplements, and honey, because their raw materials or biological sources may be exposed to contaminated soil, water, and atmospheric deposition.

Water distribution networks may also contribute to contamination with MPs. Plastic particles can be released into flowing water as a result of wear of infrastructure components made of poly(vinyl chloride) (PVC), polyethylene (PE), or polypropylene (PP). This process may be promoted by material aging, hydraulic pressure, and chemical reactions, for example with chlorine used for disinfection. Studies on drinking water quality show that MPs may still be present after treatment. Although municipal water treatment plants reduce the number of such particles, they may not remove them completely. Conventional treatment systems have been estimated to eliminate approximately 83% of this contamination [35]. Water-related contamination is more directly relevant to products that contain water or require reconstitution, such as plant-based beverages, herbal distillates, infant formulas, and enteral nutrition formulas prepared or processed with drinking water.

With the intensification of agricultural practices, the use of plastics has increased, contributing to higher levels of environmental pollution [36]. Consequently, MPs may enter health-oriented foods through contaminated soil, including contamination related to the use of agricultural films, such as mulch films and greenhouse films, as well as the application of sewage sludge and fertilizers containing processed waste [37,38,39]. The intensity of agricultural activities and the mechanization of cultivation and harvesting are also significant factors [40]. Agricultural sources are most relevant for crop-derived health-oriented ingredients produced in intensively cultivated systems. These include medicinal herbs, spices, botanical extracts, and plant-based supplement ingredients, for which plastic mulching, greenhouse production, irrigation, fertilization, and mechanical harvesting may contribute to contamination.

Water and soil pollution may lead to the contamination of plant-based raw materials. Cross-contamination with MPs can occur during cultivation, harvesting, or post-harvest processing [24]. For example, commercial cultivation of spirulina often takes place in transparent plastic tubes or shallow synthetic ponds, which may also act sources of MPs [35].

Harvesting methods for plant raw materials often involve simple systems based on sieving through nylon meshes. As a result, polyamide (PA) particles may contaminate the harvested products [41]. In addition, some producers sun-dry raw materials on synthetic fabrics, which may also introduce MPs into the product. This process may be further intensified by airborne plastic particles [25,35]. Post-harvest handling may be particularly important for medicinal herbs, herbal teas, spices, and botanical ingredients used in dietary supplements. These raw materials are often harvested, dried, sieved, and stored using materials that may release fibers or fragments before final processing.

Furthermore, animals such as bees may act as vectors for MPs [42,43]. During flight, the surface of a bee’s body can become positively charged with static electricity, causing microparticles from the environment to adhere to its hairs and wings. The accumulation of airborne plastic particles on flowers may also contribute to this process [26]. Microplastics may also originate from plastic bags used by beekeepers to feed bees powdered sugar during periods of low external food availability, such as winter or early spring [44]. In addition, densely populated areas with high levels of human activity may contribute to plastic pollution reaching beehives.

### 3.2. Processing

Microplastics may be introduced into health-oriented foods during manufacturing. Key risk points include the transport and storage of raw materials, processing, and packaging. Some of these stages require the use of specialized equipment, such as mixers, capsule filling machines, tablet presses, and packaging lines. Such equipment may release MPs during processing [24].

Before processing, raw materials may also become contaminated during handling, transport, and storage. Conveyance systems that use plastic pipelines, tubing, and filters may pose a particularly high risk because surface abrasion can release contaminants into raw materials [27]. Deposition of airborne particles may also contribute to contamination with MPs [45,46,47,48,49,50,51]. Numerous studies indicate that the dominant airborne MPs are fibers, primarily originating from synthetic textiles [52,53,54]. The type of production process is also important. Water used for washing in production facilities, including bottle cleaning, may introduce MPs into the process [29]. Furthermore, inadequately cleaned bottles may contaminate the final product with MPs [46,47,48,49,50,55,56].

In the case of honey, equipment, such as PE suction pumps, and containers may serve as sources of MPs. Beekeeping suits may also contribute to contamination. The material from which beehives are made may also be important [57].

Plastic contamination can also occur during the production of herbal distillates. The distillation process, which involves heating and condensing plant material, may inadvertently introduce contaminants into the final product [29].

Plastic particle contamination depends on the cleanliness of the production process, including the encapsulation stage [24,58]. Furthermore, mechanical operations used in processing, such as milling, mixing, and cutting, may abrade the surfaces of plastic machinery and equipment. During the production of enteral nutrition formulas, plastic filters and tubing made from various polymers may also serve as important sources of contamination with MPs [24]. Elevated temperatures may also contribute to contamination by increasing plastic degradation.

### 3.3. Packaging

Packaging may also serve as a source of MPs [57]. Potential sources of MPs can be grouped into primary packaging, secondary packaging, plastic storage containers used for raw and auxiliary materials, consumer handling, and, where relevant, clinical delivery systems. Primary packaging is in direct contact with the product and may include bottles, caps, blister cavities, sachets, and similar components. Secondary packaging, such as cartons and overwraps, is generally not in direct contact with the product but may still act as an indirect source during storage and handling. Common packaging materials, such as PE, PP, PVC, poly(ethylene terephthalate) (PET), and poly(lactic acid) (PLA), can release particles through friction and mechanical stress. MPs may be released into the product during opening, closing, squeezing, or other repeated contact with packaging, both during production and use [59,60]. Plastic containers used to store raw and auxiliary materials may also contribute to contamination with MPs [27]. Moreover, storage conditions (high temperatures and intense agitation) can accelerate plastic degradation and particle release. The type of primary packaging is also important; for example, products stored in push-through blister packs have been reported to contain lower plastic particle counts than products stored in PET bottles [51,61].

For products intended for clinical use, delivery systems should be considered separately. Feeding bottles, syringes, tubing, and infusion bags may introduce additional plastic particles during administration. The physical form of the product may also matter. Some studies suggest that powders may contain more MPs than capsules and tablets [35] However, liquid products may also be susceptible to this type of contamination [29]. In addition, temperature, packaging processes, and mechanical stress generated during transport and use may contribute to the migration of MPs into bottle contents [59,62,63,64].

### 3.4. Modes of Administration

Food-based administration routes may provide a direct pathway for the transfer of MPs into the body (Table 1) [65,66,67]. For example, some commercially available herbal tea bags contain plastic polymers or plastic-based sealing materials and therefore may represent a potential source of MPs [68]. However, the extent of particle release depends, among other factors, on brewing time [51].

The widespread use of plastics in healthcare also raises concerns about MP generation. Infusion sets, syringes, dosing lines, filters, and other components of intravenous systems are commonly made from plastics, including PVC, PE, and polyurethane (PU). During use, these materials may undergo gradual abrasion and degradation. Fluid flow through these components generates friction, and higher flow rates and longer infusion times may increase the number of particles released. In addition, prolonged storage and agitation can accelerate material aging, for example in infusion bags [69,70]. Particles collected from intravenous admixtures near their expiration dates have been shown to exhibit greater surface roughness. This suggests more advanced aging than in particles retrieved from admixtures that were not close to their expiration [71]. The composition of the infusion fluid also appears to play an important role in these described processes. Depending on the fluid type, different reactive oxygen species (ROS) may be formed. This may lead to varying degrees of material aging and particle release [70]. These findings highlight the need to monitor the cleanliness of medical equipment. The migration of such contaminants from medical devices into patients’ bloodstream may pose health risks. In this context, the release of MPs from intravenous lines used in neonates is of particular concern [72].

**Table 1:** Microplastics derived from medical devices.

Medical Device	Product Material	Test Fluid	Amount Released	Particle Size	Study Conditions	Blank Correction	Detection Method	References
Infusion bags	PP	SC, GS, GSC, MH, ES, and SBR	522–5455 MPs/L	>0.2 μm	4 °C or room temperature/kept stationary or agitated at 60 rpm	NO	µ-Raman, flow cytometry	[70]
Bottles	PP	SC	approx. 7500 MPs/L	1–62 μm	clinical settings	YES	Raman, optical microscopy	[73]
Intravenous infusion products	PVC	SC, GlS	0.36–0.72 µg	>20 nm	simulation of an actual intravenous infusion	YES	Raman, Py-GC-MS, SEM/EDX	[67]
Infusion tubes	PVC	SC, GlS, SBSo, HSSC	1003.6–3494.6 MPs (0.042–0.087 µg)	0.22–30 μm	25 °C or 37 °C	YES	Raman, TEM	[74]

MPs—microplastics; PP—polypropylene; PVC—poly(vinyl chloride); SC—sodium chloride; GS—grape sugar; GSC—glucose and sodium chloride; MH—moxifloxacin hydrochloride; ES—etimicin sulfate; SBR—sodium bicarbonate ringer; GlS—glucose solution; SBSo—sodium bicarbonate solution; HSSC—hydroxyethyl starch with sodium chloride; µ-Raman—micro-Raman spectroscopy; Raman—Raman spectroscopy; Py-GC-MS—pyrolysis-gas chromatography-mass spectrometry; SEM/EDX—scanning electron microscopy with energy-dispersive X-ray spectroscopy; TEM—transmission electron microscopy.

### 3.5. Practical Recommendations

To reduce the risk of secondary contamination with MPs during processing and packaging, regular environmental monitoring should be conducted. This monitoring should include the assessment of airborne MPs and settleable dust in production and packaging areas. It should be performed using standardized sampling procedures, appropriate field and procedural blanks, systematic recording of results, and predefined corrective actions when elevated contamination levels are detected.

Production areas should be cleaned regularly using methods that minimize particle resuspension. Ventilation and filtration systems should be inspected, serviced, and replaced promptly when they no longer meet performance standards. Potential sources of MPs from auxiliary materials, packaging components, equipment, and work clothing, particularly plastic materials and synthetic textiles, should be identified and controlled.

In addition, controlled-environment areas, such as cleanrooms or clean zones, may further reduce contamination. Strict personnel hygiene and gowning procedures should also be implemented to reduce particle shedding.

## 4. Occurrence of Microplastics in Different Product Categories

Microplastics have been identified in an increasing number of food products intended for health-related purposes [27,75,76,77]. Their occurrence in these product categories reflects the combined influence of environmental contamination, processing conditions, packaging, and administration routes.

### 4.1. Dietary Supplements

The modern market for “health-promoting” products is dominated by dietary supplements, which are defined as products containing concentrated sources of vitamins, mineral components, amino acids, fatty acids, fiber, and other ingredients intended to supplement the normal diet [24,78,79]. Although their physical forms, such as tablets, capsules, gummies, or liquids, may resemble those of medicinal products, dietary supplements are regulated as a food category in many legal systems, including those of the United States and the European Union. Consequently, they are not generally subject to the same rigorous premarket testing required for drugs [24,79,80]. Despite their widespread use, the mass production of dietary supplements poses significant challenges related to their purity and safety. In recent years, several studies have investigated the presence of MPs in different categories of dietary supplements, including spirulina products, omega-3 supplements, and fiber preparations [24,35,58,61]. Studies by Choi et al. [61], Kim et al. [58], Panneerselvan et al. [24], and Tutaroğlu et al. [35] reported the presence of MPs in all analyzed fiber and omega-3 products and in most analyzed spirulina products. Fibers were the dominant particle shape, whereas PA, PP, and PET were among the most frequently identified polymers.

A study of nine popular brands of fiber supplements purchased in Australia, including powders and gummies, detected MPs in all samples. The average intake associated with consumption of the analyzed dietary supplements was estimated at 5.89 ± 2.89 MPs/day [24]. Among the detected MPs, fibers predominated (83.4%), followed by fragments (16.6%) [24]. Because of their elongated shape, fibers may interact with biological barriers differently from fragments; however, their ability to penetrate tissues and blood–tissue barriers more deeply requires cautious interpretation [81]. Fiber sizes ranged from 120 μm to 2 mm, whereas fragment sizes ranged from 610 μm to 3 mm. The most frequently detected polymer was PA (38%). Other identified polymers included poly(diallyl phthalate) (PDAP, 21%), polyethylene–propylene diene (PEPPd, 19%), PU and PET (7% each), and PE and ethylene–acrylic acid copolymer (EAAC, 4% each). In fiber-containing supplements, black was the most common color and was detected in seven of the nine analyzed samples. All black particles were identified as PA. Among microplastic fibers, which were the dominant form of contamination in dietary fiber supplements, the most frequently observed colors after black were blue, red, white, and green, in that order (Table 2). Green particles (in the form of fragments) were rare and were recorded in only two samples. The authors also noted a possible effect of packaging, indicating that colored containers, such as red, blue, and green containers, may release more microplastic particles into the product than black, white, or silver containers [24]. The analysis also suggested a correlation between particle color and chemical composition. Black particles were identified as PA, blue particles as PET or PEPPd, red particles as PDAP, green particles as PE, and white particles as EAAC or PU [24].

In a study by Choi et al. [61] of 88 omega-3-containing products from South Korea, MPs were detected in all samples of both of animal and plant origin. The average number of particles was 16.3 ± 8.1 MPs per serving. Poly(ethylene terephthalate) (43.8%) and PP (25.2%) were the main polymers identified. Importantly, capsule oil contained three to five times more MPs than the corresponding raw oil before encapsulation, suggesting that the production process may be a major source of contamination. In another study, Kim et al. [58] found that PET and PP were the dominant polymers and together accounted for 83–95% of all identified particles. The average size of the detected MPs was 143.1 ± 193.1 μm. Particles in the 20–100 μm range were the most frequently detected, accounting for 58.5% of all particles. In the study by Choi et al. [61], fragments and fibers were the main particle shapes observed. By contrast, in the study by Kim et al. [58], the dominant size range of MPs in the analyzed omega-3 dietary supplements was 5–10 μm, accounting for 33–40% of all particles. Particles in the 5–20 μm range accounted for 60–73% of all detected MPs [58,61] (Table 2). The presence of PP and PET particles, which are commonly associated with plastic packaging components such as bottles and caps, suggests that factory infrastructure, packaging, and plastic components of production lines may be important sources of MPs in omega-3 supplements [61].

In a study of spirulina-containing supplements, 29 products were analyzed. MPs were detected in 26 products (89.6%) [35]. The average content was 13.77 ± 2.45 MPs/100 g dw. Spirulina powder tended to be more contaminated (17.34 ± 4.22 MPs/100 g) than capsules or tablets (10.43 ± 2.45 MPs/100 g). The most frequently identified polymer was PP (31.6%) [35]. Other commonly detected polymers included polystyrene (PS; 8.3%) and PE (8.3%), polyester (PES; 6.25–7.8%), and poly(vinylidene chloride) (PVDC; 6.25%) [35]. In total, ten types of synthetic polymers and cellulose were identified in the analyzed spirulina samples. Materials such as nylon-6, PU, acrylonitrile-butadiene-styrene (ABS) copolymer, and styrene-acrylonitrile copolymer were also detected in smaller amounts, each accounting for approximately 2.08%. The predominance of PP may be related to its widespread use in packaging, including large woven bags used to transport bulk food products, such as spirulina. In turn, the presence of PE may be linked to its broad use in food packaging [90]. Fibers were the predominant MP shape (61.7%), whereas fragments accounted for the remaining 38.3%. Fragments ranged in size from 0.07 to 2.15 mm, whereas fibers ranged from 0.19 to 5.69 mm [35]. The most common color of MPs in the analyzed spirulina-containing dietary supplements was blue, accounting for more than half of all identified particles (52.8%). The remaining colors were black (25.4%), white (10.9%), and other colors (10.9%), including red, purple, gray, green, brown, yellow, and transparent particles (Table 2). The variation in color and polymer composition suggests that the sources of contamination are diverse and may include the water used in production [91], processing equipment, and final packaging [92].

### 4.2. Herbs and Plant-Based Beverages

Plant-based products, such as loose spices, fresh leafy vegetables and herbal infusions, form complex food matrices that, because of their structure and processing, may be particularly susceptible to the accumulation of MPs [25,51,82]. Studies on common loose spices, including turmeric, black pepper, and chilli, have confirmed the presence of MPs in all analyzed samples, with concentrations ranging from 500 to 1100 MPs/kg. Spices sold in bulk consistently showed higher contamination levels than products in sealed packaging [25]. A similar pattern has been observed in herbal infusions and distillates [51,93].

Polyethylene and PP predominated in loose spices, most likely due to the use of plastic bags and handling equipment in retail settings. Fibers were the dominant morphological form, accounting for 60–70% of the identified particles. Their prevalence suggests possible contamination via atmospheric deposition, for example from textiles [94]. The study also identified fragments, which accounted for approximately 25–35.6% of the particles (Table 2). Their presence is usually associated with mechanical abrasion of plastic surfaces, machinery, or packaging [95]. Films and granules occurred less frequently and complemented the morphological profile, particularly in samples sold in bulk [25]. Most MPs in spices were very small, with more than 90% of particles smaller than 100 µm. In terms of color, white and black predominated, possible contributions from typical packaging materials, such as transparent bags or black caps. However, a greater variety of colors, including blue, red, and yellow, was observed in factory-packaged products, which may reflect more complex industrial processes [25]. The main sources of MPs in loose spices may vary depending on the form in which they are sold, that is, in bulk or packaged. Spices sold in bulk consistently showed higher contamination with MPs than factory-packaged products. This suggests that exposure to airborne dust in open markets and frequent product handling may be important routes of contamination [25,94]. Analysis of polymer composition confirmed that PE and PP predominated in loose spices, which may be linked to the use of plastic bags and utensils [25], whereas PET and nylon were more commonly found in packaged spices and may be associated with production lines and sealed packaging [95].

Contamination of leafy vegetables, such as lettuce, chives, and cilantro, with MPs has been reported in studies conducted in the Brazilian Amazon [82]. Because of their surface structure and cultivation conditions, these products may accumulate polymer particles. In the study Staffen et al. [82], MPs were detected in all examined vegetable species. The frequency of contamination was highest for lettuce and chives (90% of samples), whereas for cilantro it was 76.6%. The size of MPs in leafy vegetables varied widely; the smallest particles identified were 67 μm, and the largest reached 4.865 mm, with both extreme values recorded in lettuce samples. Fibers were the dominant form, accounting 95.1% of all identified particles. Fragments constituted only a small proportion of the contaminants (4.9%). The most frequently recorded color was blue (83.8%), followed by red (Table 2). White, purple, gray, green, and transparent particles were less common. However, the study did not identify the polymer types.

The presence of MPs in bottled herbal distillates, also known as hydrosols or herbal waters, has been reported in studies of popular retail brands [29,93]. Pirsaheb et al. [29] showed that 92% of the analyzed herbal distillate samples were contaminated with MPs. The average concentration of MPs in these products was 7.32 ± 9.24 MPs/L. Contamination levels varied considerably depending on the brand; some products contained an average of 26.4 MPs/L, whereas others contained only 2.8 MPs/L (Table 2). The authors suggested that PET bottles may be an important source of PET MPs in these products. Fourier-transform infrared spectroscopy (FTIR) analysis also identified PA. The main forms of MPs were fibers (62.30%) and fragments (37.70%). Fibers may originate from water used in the production process or from synthetic textiles, whereas fragments may result from the degradation of plastic packaging. Small MPs predominated. More than half of the particles (56.56%) were smaller than 500 μm. As particle size increased, their frequency of occurrence decreased—particles measuring 4000–5000 μm accounted for only 3.55% of the contamination. The most common particles were transparent (29.23%), white (23.50%), and black (16.12%). This color distribution may correspond to the colors of the bottles and caps used in the herbal distillate industry [29].

Contamination of herbal teas with MPs may represent a food safety concern because these products are widely consumed for their perceived health-promoting properties [51,68]. Studies of popular packaged teas, such as linden, sage, chamomile, and green tea, revealed the presence of synthetic particles in all but one of the analyzed brands. The average concentration of MPs in the infusions was 4 ± 1.01 MPs/mL, with the highest amount recorded in linden tea [51]. The analysis identified three main types of plastic: PET, which accounted for 60% of the contaminants; ethylene-vinyl acetate copolymer (EVA), which accounted for 35%; and polyacrylonitrile (PAN), which accounted for 5%. Poly(ethylene terephthalate) is particularly common because it is frequently used in plastic tea bags and outer packaging. Morphologically, all identified particles in herbal teas were fibers [53,96]. The prevalence of this form suggests possible atmospheric deposition, for example from synthetic textiles, during production, as well as abrasion of packaging materials [53,54]. Most of the detected fibers were small. The average size was approximately 191.82 μm, and 95% of all particles were shorter than 500 μm. The most common length range was 0–100 μm. The color profile was dominated by dark blue (65%) and red (35%) fibers (Table 2). The main mechanism by which MPs enter the infusions appears to be the migration of particles from synthetic tea bag materials directly into water during brewing [51,97].

### 4.3. Honey

Honey, traditionally regarded as a product of high purity with valued health-promoting properties, has unexpectedly been identified as a reservoir of plastic pollutants [83,86,98,99]. The presence of MPs in honey is of particular concern from a food safety perspective because honey is typically consumed raw, without any prior processing that could remove contaminants [86,100].

Studies conducted in various parts of the world have shown that honey can be contaminated with MPs, although contamination levels vary considerably depending on the region, degree of urbanization, and bee species [26,57,86,87,101]. An analysis of available sources indicates that the regions with particularly high levels of honey contamination are Malaysia, Indonesia, Brazil, and Turkey. Because studies report the abundance of MPs using both mass-based and volume-based units, direct comparisons are difficult. However, when mass-based and volume-based data are considered separately, regions with relatively high numbers of MPs in honey can be identified.

Malaysia showed some of the highest levels, particularly in honey from stingless bees (*Heterotrigona itama*), in which the average microplastic content was 8.18 ± 2.57 MPs/g, equivalent to more than 8000 MPs/kg [57]. By comparison, honey from the European honeybee (*Apis mellifera*) in the same country showed lower values, averaging 5.52 ± 1.13 MPs/g [57]. In the study by Basaran et al. [85] the average concentration of MPs in honey from Turkey was 314 ± 353 MPs/kg. However, samples from heavily industrialized and densely populated regions, such as Tekirdağ, contained up to 1280 MPs/kg. Compared with Malaysia or Turkey, honey samples from Western Europe, including Germany, France, Italy, and Spain, and from Kosovo showed lower or moderate levels of contamination with MPs. Studies published by Liebezeit and Liebezeit [44] reported an average microplastic content of approximately 166–175 MPs/kg in honey from Western Europe. A lower number of MPs (124 ± 68.3 MPs/kg, with a maximum of 360 MPs/kg) was recorded in honey from regions with intensive industrial development, such as Gjilan and Vitia (Kosovo) [26].

In Indonesia, in the Meratus Geopark region, analyses of bee products revealed high levels of contamination, ranging from 3090 to 3180 MPs/L in urban areas, whereas a value of 3620 MPs/L was recorded at one rural location, Kelulut Dua Puteri [87]. Compared with honey from Brazil, produced by *Melipona quadrifasciata* bees in the São Paulo area, Indonesian honey showed higher levels of contamination with MPs (Table 2). Brazilian honey samples contained an average of 1450 MPs/L, with the most contaminated samples reaching up to 2600 MPs/L [83]. By contrast, lower contamination levels were found in honey from Ecuador. The product showed relatively low contamination levels, averaging 54–67 MPs/L [84].

The prevalence of these polymers in honey suggests that this product may serve as a useful indicator of land-based plastic pollution. The presence of specific polymer types in honey may be associated with local plastic production, packaging practices, and the equipment used in apiaries [26]. The most commonly detected polymers include PE, PP, PET, EVA, and PA. Polyethylene is one of the most common contaminants found in honey from Kosovo, Turkey, Ecuador, Brazil, and Malaysia [26,57,83,84,85]. In the study by Başaran et al. [85], PE accounted for up to 62% of all identified MPs, and its presence may be linked to honey extraction methods and packaging. Polypropylene is frequently found alongside PE and is regularly identified in bee products. In honey from Brazilian stingless bees (*Melipona quadrifasciata*), PP was the dominant polymer, accounting for 52.9% of the identified particles [83]. PET is commonly found in both artisanal and industrial honey, and plastic packaging and synthetic fabrics have been suggested as possible sources [86,98]. PET is commonly found in both artisanal and industrial honey, and plastic packaging and synthetic fabrics have been suggested as possible sources [86,98]. In studies conducted in Kosovo [26] and Turkey [86], EVA was often the most common polymer. This polymer is widely used in beekeeping pipes and packaging [102]. Nylon-6, a commonly detected polymer in honey, is often attributed to beekeepers’ protective clothing and specialized mesh used in hives [85]. Because of their shape, fibers were the predominant form of particulate matter in most studies, often accounting for more than 90% of all particles, for example in Kosovo and Malaysia [26,57]. However, in some regions, such as Turkey, fragments predominated, accounting for up to 95%, which may be linked to the degradation of plastic beekeeping equipment [85,103]. Films, foams, and granules were less common. The analyzed particles typically ranged from 20 µm to 5 mm in size [26,57,83]. Exceptions to this size range were reported for honeys from Ecuador, Brazil, and Western Europe [43,44,84,85]. The color spectrum was highly diverse. The most frequently identified particles were black, blue, and transparent/colorless (Table 2). Red, green, brown, yellow, and purple particles were also detected.

### 4.4. Infant Formulas

Infant formulas constitute complex food matrices designed as substitutes for breast milk. They are aqueous-emulsion matrices containing fats, proteins, carbohydrates, vitamins and mineral components. In products intended for infants with digestive issues, functional additives, such as thickeners, may also be used. This multicomponent nature is analytically important because it complicates matrix digestion and increases the risk of signal interference during plastic identification. From the perspective of biological interactions, the potential formation of a “protein corona” on the surface of plastic particles should also be considered.

Studies indicate the widespread presence of MPs in infant formulas [75,76,77,88]. Kadac-Czapska et al. [76] demonstrated the presence of such contaminants in all tested starter infant formulas sold in Poland, with an average concentration of 42 ± 27 MPs/100 g of product. The detected particles ranged in size from 6 µm to 4.4 mm. Most identified particles were fibers, fragments, and films, with black, blue, and white being the dominant colors. The most common polymers were PA, PE, PP, and PET. Across product types, the highest average concentration was recorded for standard formulas (55 ± 26 MPs/100 g), followed by anti-reflux formulas (41 ± 30 MPs/100 g), hypoallergenic formulas (23 ± 19 MPs/100 g), and “comfort” formulas (22 ± 11 MPs/100 g) [76]. Data for follow-on formulas (6–12 months) show a similar pattern of widespread contamination, with an average concentration of 55 ± 19 MPs/100 g [77]. In these formulas, particle lengths ranged from 30 µm to 4.5 mm, and widths ranged from 6 to 268 µm were reported. Compared with starter infant formulas, a similar set of polymers was observed, but with greater morphological diversity, including fibers, fragments, films, and foams. Black, blue, and red particles were the most prevalent [77]. In a cross-sectional study from Turkey, MPs were also detected in 100% of samples, with an average concentration of 31.3 MPs/100 g. In that study, fibers predominated, followed by fragments and films. In a similar study conducted in China, Zhang et al. [88] found an average of 5 ± 3 MPs/100 g, although products in cartons exhibited significantly higher values (7 ± 3 MPs/100 g) than those in cans (4 ± 3 MPs/100 g). The main polymers identified were PE, PET, PP, PA, and PVC. This study indicated that packaging type may influence the polymer profile. Fragments and fibers predominated in the samples, and most particles were <50 µm in size [75].

A compilation of the results suggests that differences between markets may relate to both particle counts and size distributions and may also reflect different limits of detection and analytical protocols. Comparisons between the presented findings therefore require caution. Variations in the counts of MPs may result not only from actual contamination levels but also from methodological differences, including the pore size of filters used for isolation, particle counting methods, and polymer identification techniques, such as µ-Raman or µ-FTIR.

The potential health effects associated with infant exposure to MPs are not yet fully understood, but these particles may act as carriers of contaminants or trigger an inflammatory response. Therefore, practical recommendations include reducing MPs at the production stage, for example by using filters to remove MPs from raw milk, minimizing the use of plastic components during processing, and exercising caution when preparing formula for consumption. Manufacturers are also advised to monitor plastic particles in ambient dust, as airborne particles may constitute an important source of contamination during production. Regulatory and inspection authorities should consider introducing guidelines for monitoring MPs in infant products and conducting toxicological studies to assess their potential risks.

### 4.5. Clinical Nutrition Products

Clinical nutrition products are characterized by highly specific compositions. This group includes enteral and parenteral nutrition formulas.

Enteral nutrition formulas are specialized products designed to provide nutrients, such as proteins, carbohydrates, fats, vitamins, and mineral components, to patients who are unable to consume adequate meals [104,105]. They are primarily sold in liquid form [106,107]. The use of enteral nutrition formulas is determined based on the patient’s health status and nutritional needs, and their administration occurs under specialist supervision and according to specialist recommendations [108].

Başaran et al. [27] demonstrated that enteral formulas may be contaminated with MPs; the average particle concentration was 45 ± 63 MPs/L of prepared solution. Fibers predominated over fragments, and the most common color was black, followed by blue and orange. The particle size range was 10 µm–2.1 mm, with an average size of 548 ± 526 µm. The authors estimated patients’ intake of these particles at approximately 30–76 MPs/day.

Parenteral nutrition solutions, in turn, are macronutrient preparations containing glucose, amino acids, and lipid emulsions. They also contain electrolytes and vitamins [72]. However, intravenous therapy has been identified as a potential route by which MPs may enter the bloodstream [89].

A study by Vercauteren et al. [72] analyzed the migration of MPs in a model of intravenous infusion in neonates. The authors examined a lipid emulsion and a crystalloid solution used in neonatal intensive care. In the lipid emulsion, 0.8 MPs/mL were detected, whereas lower concentrations were found in the crystalloid solution. The results indicated that PP, likely originating from the equipment used, was the most frequently detected polymer in the lipid emulsion, whereas PET predominated in the crystalloid solution. Most particles ranged in size from 25 to 175 µm.

The high fat and protein content of clinical nutrition products may contribute to the deposition or adsorption of MPs onto food matrix components. Furthermore, high concentrations of these components increase the viscosity of the aqueous phase, thereby complicating filtration. The presence of salts and amino acids may also promote aggregation of MPs, limiting their transport through filters. Consequently, isolation requires robust matrix digestion methods. The complexity of the matrix may also generate a substantial organic background, which can hinder analysis using spectroscopic techniques. Despite these analytical challenges, clinical nutrition products should be subject to rigorous quality control with regard to plastic particle content. The introduction of standardized analytical methods is recommended, including minimum detection limits, matrix digestion procedures, and mandatory procedural blanks. Furthermore, toxicological studies focused on the exposure of adults and children, especially neonates, to MPs during clinical nutrition are necessary to evaluate the potential health effects associated with these contaminants.

## 5. Exposure Assessment

Published studies offer various methods for assessing human exposure to MPs in food products. These metrics include the “Predicted Daily MPs Exposure” (PDME), which estimates daily exposure per suggested serving [24]. Other indices include “MPs Contamination Factors” (MCF/MPCF), the “MPs Pollution Load Index” (MPLi), and the “MPs Polymer Risk Index”—also referred to as the “Polymeric Hazard Index” (PHI) [24,51]. Because researchers report these metrics in various forms, such as numerical values (1–10,000), verbal ratings (low, moderate, significant, very high), or categorical levels (I–V), direct comparisons across studies remain challenging [24,26,27,51]. Furthermore, many studies have not yet incorporated these metrics into their MP exposure assessments [61,72,76]. It is important to distinguish between occurrence, exposure, hazard, and risk. Occurrence refers to the presence and abundance of MPs in a given product or matrix. Exposure describes the amount of MPs that may be ingested over a defined period, for example, as estimated by EDI or EAI. Hazard refers to the potential of MPs to cause adverse effects, which may depend on particle size, shape, polymer type, chemical additives, and other associated contaminants. Risk, in contrast, requires the integration of exposure and hazard information [24,26,27,51]. PHI, or Polymer Hazard Index, is an indicator that incorporates polymer-specific hazard scores to provide a relative estimate of the potential hazard associated with detected MPs. However, PHI should not be interpreted as a direct measure of human health risk because it does not fully account for particle size, morphology, dose, route of exposure, additives, or biological persistence [27]. Some indicators describe occurrence or contamination levels, while others estimate intake. Therefore, this review focuses mainly on estimated daily intake (EDI) and estimated annual intake (EAI), which describe the number of MPs ingested from food over defined timeframes. For human health risk assessment, exposure estimates would need to be compared with health-based guidance values or acceptable exposure thresholds for MPs; however, such values have not yet been globally established. Table 3 presents a comparison of exposure to MPs from different food products intended for health-related purposes.

### 5.1. Exposure Scenarios for MPs

Dietary supplements are widely used by both healthy individuals and people with health conditions across different age groups. To date, only a limited number of studies have investigated the presence of MPs in dietary supplements. The available data suggest that exposure to MPs from fiber supplements and omega-3 products (Table 3) [24,61] is lower than exposure from staple foods. For example, Samandra et al. [109] estimated that the Australian population is exposed to approximately 400 MPs per year through bottled water consumption [109]. Moreover, because supplements are often consumed with water, beverages, or food, their use may contribute to cumulative exposure to MPs [24].

Herbs and spices are generally consumed infrequently and in small quantities. Gökkaya et al. [51] reported that, due to their comparatively lower body weight, women may have higher exposure to MPs from tea than men. The authors also observed that individuals aged over 65 years consumed more herbal tea than other age groups, which may lead to increased exposure to MPs. Jahedi et al. [25] stated that their scenario-based estimate of exposure to MPs from spices (188–613 MPs/person/year) was intended to provide contextual information on potential exposure rather than a direct assessment of health risk. Therefore, determining the health implications of MPs ingestion from these products remains challenging.

Honey is an example of a food product whose consumption depends on culture, geography, and individual dietary habits [26]. Infants younger than 12 months are not included in assessments of exposure to MPs from honey, as its consumption is not recommended for this age group because of the risk of botulism [110]. In contrast, older children are believed to consume more honey relative to body weight than adults, which may result in higher exposure to MPs [86]. It should also be emphasized that honey may be added to other food products and dietary supplements, making assessments of exposure to MPs more complex [111]. Importantly, Bilecen and Altunışık [86] observed higher exposure to MPs from artisanal honey than from industrial honey, which may result from differences in processing, storage, or packaging.

Infant formula is another category of foods intended for health purposes. Infants are particularly vulnerable to exposure to MPs from food because of their higher intake per kilogram of body weight and their developing metabolic and physiological systems [77]. International health guidelines recommend exclusive breastfeeding for the first six months of life [112]. When breastfeeding is not possible, infant formula is used as the primary source of nutrients. Therefore, contamination of infant formula with MPs may represent a potential exposure concern for infants. However, infants may also be exposed to MPs from several other sources during feeding. For example, water used to prepare formula may contain plastic particles. Microplastics may also be released from plastic containers, including feeding bottles [76]. Another challenge is the lack of comparisons between exposure to MPs in infants younger than 6 months and in older children with more diverse diets. Older children may have higher exposure to MPs from a broader range of foods, but they also have a more developed immune system. In addition, the digestive systems of infants and older children differ substantially, including in gastric pH, stomach capacity, gut microbiota composition, and intestinal permeability [113].

Clinical nutrition is a specialized form of nutrition used when patients cannot meet their nutritional requirements through a regular diet. Vercauteren et al. [72] observed that exposure to MPs may vary depending on the type of nutrition, as lipid preparations may promote the release of MPs from plastic medical materials. Another issue is that patients receiving clinical nutrition may have dysfunction in specific sections of the gastrointestinal tract. Further research is needed to compare the effects of exposure to MPs delivered to the stomach with those of exposure delivered directly to the small intestine. Moreover, Chen et al. [114] suggested that MPs may enter the bloodstream through infusion tubes and blood collection needles. Overall, evaluating exposure to MPs during clinical nutrition is complex because nutrition formulas may become contaminated during manufacturing and MPs may also be released from plastic medical devices. The duration of clinical nutrition should also be taken into account.

For foods intended for health purposes, exposure should not be considered only for individual products. In practice, individuals may use several of these products at the same time, for example, dietary supplements together with herbal products or fortified foods, whereas infants or patients may be exposed through infant formula or clinical nutrition products [24,51,61,76,77]. This means that MPs from different sources may contribute to the same overall dietary exposure. A similar idea is used in combined exposure scenarios in other areas of risk assessment, including plant protection products [115]. This issue may be especially important for groups that use certain products repeatedly or over long periods. Infants may consume infant formula as their main or only source of nutrition [76,77]. Patients may depend on clinical nutrition products for weeks or months, and older adults may take several dietary supplements or other health-oriented products at the same time [24,61]. Therefore, future exposure assessments should look beyond plastic concentrations in single products. Serving size, frequency of use, duration of exposure, and the possible combined use of several product categories should also be taken into account [24,51,116,117,118].

### 5.2. Health Implications

The main toxic effects associated with exposure to MPs include inflammation, oxidative stress, endoplasmic reticulum stress, apoptosis, and alterations in autophagy [119]. The gastrointestinal system is likely to be the primary site of exposure to MPs when foods intended for health purposes are consumed [120]. The health implications of MPs depend on the characteristics of the plastic particles, including their morphology, size, and chemical composition. Further research is needed to establish reference doses that would support human health risk characterization for MPs. Particle size should be considered when deriving such doses, together with morphology, polymer type, exposure level, and route of exposure [121]. The lack of such size-informed reference doses remains a major gap in exposure and risk assessment. Smaller MPs are often expected to cause stronger biological responses than larger particles, partly because they may be more readily taken up by cells, cross biological barriers, and reach different tissues [122]. According to the World Health Organization (WHO), particles larger than 150 µm are less likely to be absorbed after ingestion [123].

High exposure to MPs has been suggested to be associated with gut-related disturbances, particularly in children. Ke et al. [124] reported that the abundance of bacteria from the genus *Alistipes* in the gut microbiota was higher in children with low levels of MPs. Baek et al. [125] suggested that Korean red ginseng extract may reduce intestinal accumulation of MPs by protecting the intestinal epithelial mucosal barrier. This finding suggests a potential protective effect against intestinal MPs accumulation, although further studies are needed to confirm its relevance in humans. Wu et al. [126] also proposed a possible link between MPs and thrombosis, which may be relevant for patients receiving parenteral nutrition.

A major challenge in assessing the health implications of MPs is that the available evidence comes from different types of studies. These include in vitro tests, animal studies, human biomonitoring, and small-scale epidemiological research. The studies also differ in the particle sizes and polymer types examined, experimental model, dose, and route of exposure. For this reason, effects such as inflammation, oxidative stress, endoplasmic reticulum stress, changes in autophagy, cell death, microbiota disruption, bloodstream transport, and tissue distribution should not be interpreted as equally well established for dietary exposure in humans.

## 6. Sample Preparation, Analytical Methods, and Standardization

It is important to distinguish between MPs that may enter products during production, processing, contact with equipment, transport, or packaging and particles introduced during sample handling and laboratory analysis. When evaluating plastic particles in products with potential health relevance, it is necessary to consider the sample preparation procedure, contamination control strategy, particle size cut-off, and analytical technique used. This distinction is especially important for complex matrices such as infant formula and clinical nutrition products, where sample composition can interfere with particle isolation and identification. These matrices often contain high levels of lipids, proteins, carbohydrates, and mineral components, which can make digestion and filtration more difficult, increase the organic background, promote particle loss or agglomeration, and reduce the reliability of spectroscopic identification. Laboratory contamination is a major concern in MPs analysis. Airborne fibers, laboratory clothing, reagents, water, consumables, glassware, and analytical equipment can all introduce particles into a sample and may lead to overestimation. Therefore, quality assurance and quality control measures should be applied to each analytical batch. These measures include procedural blanks, field, or laboratory blanks when appropriate, filtered reagents, covered glassware, clean-air conditions, limited use of plastic materials, and low-shedding laboratory clothing, such as cotton lab coats. Restricting access to the analytical area and clearly documenting contamination control procedures can further improve data reliability.

Effective digestion, density separation, filtration, and isolation procedures, along with recovery experiments using representative polymer types, particle sizes, and shapes, are crucial for assessing method performance. Visual inspection and optical microscopy alone are not sufficient for reliable polymer identification because they cannot confirm the chemical composition of suspected particles. Therefore, these methods should be combined with spectroscopic techniques, especially FTIR/µ-FTIR or Raman/µ-Raman spectroscopy [2,5,121]. Raman/µ-Raman spectroscopy can provide excellent spatial resolution and may enable the detection of microparticles that are difficult to analyze using conventional FTIR-based techniques. However, its performance may be limited by fluorescence, pigments, additives, and matrix residues. FTIR and µ-FTIR are widely used for polymer identification and are considered reliable for particles of sufficient size [2,5]. Their application to very small particles, however, remains challenging and depends on several factors, including instrumental configuration, measurement mode, substrate, and spectral quality. Therefore, direct comparisons between studies and product categories require appropriate sample preparation, validated recovery, clearly defined lower size limits, consistent particle counting rules, and transparent reporting of analytical parameters.

In this context, current and emerging International Organization for Standardization (ISO) standards may help improve the comparability of microplastic analyses, although food matrices still require more specific guidance. ISO 24187:2023 provides general principles for the analysis of MPs in environmental matrices, including sampling, sample preparation, and the determination of representative sample quantities [127]. The working draft ISO/WD 25654 addresses reference materials for the validation of microplastic detection methods and specifies top-down procedures for producing irregularly shaped thermoplastic polymer powders with the longest particle dimension between 10 µm and 1000 µm [128]. Moreover, ISO 16094-2:2025 outlines principles for the analysis of MPs in drinking water and waters with low suspended-solids content using microscopy coupled with vibrational spectroscopy [129]. However, similar standardized and matrix-specific procedures are still needed for food and health-related nutritional products, including guidance on sampling equipment, sample mass, digestion and isolation conditions, contamination control, recovery assessment, polymer identification, and reporting criteria.

## 7. Conclusions

Scientific studies have demonstrated that MPs can contaminate foods intended for health purposes. The detected MPs vary in their physical and chemical characteristics, suggesting that they may enter food through different pathways. Infant formula and clinical nutrition products require particular attention because they are intended for vulnerable populations. Available experimental evidence suggests that MPs may adversely affect biological systems; however, major uncertainties remain regarding dose metrics, human exposure, and clinically relevant health outcomes. Assessing human exposure to MPs from food is complex and requires consideration of several factors, including age, health status, food type, frequency of consumption, packaging, and the use of feeding aids.

To date, most studies have estimated human exposure to MPs based on the number of particles detected in food. However, exposure estimates remain uncertain because MPs contamination in food products is not constant and may be influenced by several factors, including production, packaging, transport, food preparation, heat treatment, and serving method. Therefore, accurately determining human exposure to MPs from food consumption remains challenging. Further toxicological studies are needed to establish health-based guidance values for human exposure to MPs below which adverse health effects are not expected. Products intended for vulnerable groups also require more detailed investigation. In the future, routine monitoring and clear regulatory guidance for these products could help improve consumer protection.

This review has several limitations. Available studies differ in sample preparation, analytical methods, size thresholds, contamination control, and reporting units, making direct comparisons difficult. Another important limitation is the lack of standardized procedures for the analysis of MPs in food products. In addition, some product categories, especially those intended for vulnerable groups, are still represented by a limited number of studies. Together, these limitations make it difficult to estimate human exposure to MPs from health-oriented foods and prevent a full risk characterization. Further research and routine monitoring could help improve data comparability and support consumer protection.

## Figures and Tables

**Table 2:** Detected microplastics in food products intended for health-related purposes.

Product	Polymer	Quantity/Concentration	Particle Size	Shape	Color	Detection Method	References
Dietary fiber supplements	PA, PDAP, PEPPd, PU, PET, PE, EAAC	5.89 ± 2.89 MPs/day	120 µm–3 mm	fibers and fragments	black, blue, red, green, and white	µ-FTIR; SEM/EDX	[24]
Omega-3 oil supplements	PP and PET	raw oil: <2.2 ± 1.7 MPs/g; capsule oil: <10.6 ± 8.9 MPs/g	>5 µm	-	-	µ-Raman; µ-FTIR	[58]
Omega-3 products	PET, PP, PE, PMMA, nylon, and PU	9.5 ± 5.3 MPs/g; 16.3 ± 8.1 MPs/serving	>20 µm	fibers and fragments	-	µ-FTIR	[61]
Packaged spirulina products	PP, PS, PE, PES and 10 synthetic polymers + cellulose	13.77 ± 2.45 MPs/100 g dw	70 µm–5.7 mm	fibers and fragments	blue, black, white, and others	microscopy; µ-Raman	[35]
Spices (turmeric, red pepper/chili, black pepper)	PE, PP, PET, and nylon	turmeric bulk 880 ± 130 MPs/kg; turmeric packaged 800 ± 250 MPs/kg; red pepper bulk 860 ± 80 MPs/kg; red pepper packaged 760 ± 160 MPs/kg; black pepper bulk 520 ± 40 MPs/kg; black pepper packaged 620 ± 70 MPs/kg	<100 µm	fiber, granules, and films	white, black, blue, red, and yellow	stereomicroscopy; Raman; SEM	[25]
Bottled herbal distillates	PET and nylon	7.32 ± 9.24 MPs/L	<500 µm	fibers and fragments	transparent, white, black and other	stereomicroscope; fluorescence microscopy; SEM; FTIR	[29]
Packaged herbal teas	PET, EVA, and PAN	0–12 MPs/mL	35.67–604.87 µm	fibers	dark, blue, and red	stereomicroscopy; ATR-FTIR	[51]
Plant-based foods (lettuce, chives, cilantro)	-	247 MPs	67 µm–4.9 mm	fibers and fragments	blue, white, purple, grey, green, and transparent	microscopy	[82]
Honey	EVA, PE, nylon-6, PET, and PP	124 ± 68.3 MPs/kg	20–541 µm	fibers and fragments	black, blue, transparent, green, red, brown, and purple	stereomicroscope; ATR-FTIR	[26]
Honey (stingless bee, *Heterotrigona itama* and honeybee, *Apis mellifera*)	LDPE	8.18 ± 2.57 MPs/g (stingless bee, *Heterotrigona itama*); 5.52 ± 1.13 MPs/g (honeybee, *Apis mellifera*)	700 µm–5.0 mm	fibers and fragments	black, red, blue, purple, brown, and yellow	stereomicroscope; SEM imaging; FTIR	[57]
Honey	PP, PET, PE, and PS	0.1–2.6 MPs/mL	≥50 µm	fibers and other	transparent	stereomicroscopy; µ-Raman	[83]
Honey	PE, PP and PAA	industrial honey: 54 MPs/L; craft honey: 67 MPs/L	2.48 µm–6.7 mm	fibers and fragments	-	FTIR	[84]
Honey	PE, PP, EVA, and nylon-6	314 ± 353 MPs/kg	133 µm–20 mm	fibers and fragments	black, brown, green, red, yellow, and transparent	microscopy; FTIR	[85]
Honey (industrial vs. artisanal)	EVA, PET, PE, PA, and HFFR	79.3 ± 13 MPs/kg;	85 µm–1.2 mm	fibers and fragments	blue, black, red, and green	stereomicroscopy; ATR-FTIR	[86]
Honey	*-*	166 ± 147 MPs/kg	~10 µm–~9 mm	fibers and fragments	transparent	dissection microscopy	[44]
Honey, pollen, propolis	*-*	60 MPs/100 mL (rural-yard honey); 37 MPs/100 mL (urban-yard pollen); 49 MPs/100 mL (rural-yard propolis)	~25.8–154.1 µm	fibers, fragments, filaments, and foams	*-*	stereomicroscopy	[87]
Honey	-	2–336 MPs/kg	>40 µm	fibers and fragments	-	dissection microscopy	[43]
Infant formula	PE, PET, PP, PA, PVF, PVAc, PVC, PVS, and SAC	31.3 MPs/100 g	95 µm–4.8 mm	fragments, fibers, and films	black, blue, red, white, yellow, transparent, and gray	stereoscopic microscopy; µ-Raman	[75]
Infant formula	PE, PET, PP, PA, and PVC	5 MPs/100 g	mostly <50 µm	fragments, fibers, and films	-	FTIR	[88]
First infant formula	PA, PE, PP, PET, PAA, PAN, PC, and SBS	42 MPs/100 g	6 μm–4.4 mm	fibers, fragments, and films	black, blue, white, brown, transparent, red, yellow, and green	optical microscopy; µ-Raman	[76]
Follow-on infant formula	PA, PE, PP, PET, PAA, PS, PAN, and PC	55 MPs/100 g	6 μm–4.5 mm	fibers, fragments, films, and foams	black, blue, red, brown, green, yellow, and transparent	optical microscopy; µ-Raman	[77]
Enteral nutrition formulas	PMMA, PET, PEVA, PA, and PU	45 MPs/L	10 μm–2.1 mm	fibers, and fragments	black, blue, orange, green, red, grey, and multicolor	stereoscopic microscopy; µ-Raman	[27]
Crystalloid solutions	PET	˂0.8 MPs/mL	25–175 μm	-	-	FTIR	[72]
Lipid emulsion	PE	0.8 MPs/mL	25–175 μm	-	-	FTIR	[72]
Intravenous admixtures	PE-*co*-PP, PP, PVP, and PU	49–750 MPs/L	˂100 µm	fibers and fragments	-	stereoscopic microscopy; µ-FTIR	[71]
Sodium chloride injections	PE, PP, PE-*co*-PP, PET, ABS, and EPDM	2.91 MPs/L	15.61 µm–1.2 mm	fibers and fragments	-	µ-FTIR	[89]
Hypertonic fluid	PE, PET, PA, PS, PVC, POM, PP, PVDC, PMMA, PU, PVAc, and rubber	62.82 MPs/L	40 µm–2.4 mm	fibers and fragments	transparent, blue, gray, red, and black	stereoscopic microscopy; µ-Raman	[65]
Sodium chloride solution	PE, PA, PC, and PS	0–2 MPs/L	4–148 µm	fragments	transparent	optical microscopy; µ-Raman	[66]
Intravenous Infusions	PP, PE, PA, PU, PET, SBR, PEVA, and PTFE	9–299 MPs/L	˂100 µm	fibers and fragments	-	stereoscopic microscopy; µ-FTIR	[69]

MPs—microplastics; PA—polyamide; PDAP—poly(diallyl phthalate); PEPPd—polyethylene-polypropylene diene; PU—polyurethane; PET—poly(ethylene terephthalate); PE—polyethylene; EAAC—ethylene-acrylic acid copolymer; PP—polypropylene; PMMA—poly(methyl methacrylate); PS—polystyrene; PES—polyester; EVA—ethylene-vinyl acetate; PAN—polyacrylonitrile; SBS—styrene-butadiene copolymer; LDPE—low-density polyethylene; PAA—poly(acrylic acid); HFFR—halogen-free flame retardants; PVF—poly(vinyl fluoride); PVAc—poly(vinyl acetate); PVC—poly(vinyl chloride); PVS—poly(vinyl sulfonate); SAC—poly(styrene-co-acrylonitrile); PC—polycarbonate; PEVA—poly(ethylene-co-vinyl acetate); PE-co-PP—poly(ethylene-co-propylene); PVP—polyvinylpyrrolidone; ABS—poly(acrylonitrile-co-butadiene-co-styrene); EPDM—ethylene propylene diene monomer; POM—polyoxymethylene; PVDC—poly(vinylidene chloride); PTFE—poly(tetrafluoroethylene); µ-FTIR—micro-Fourier transform infrared spectroscopy; SEM/EDX—scanning electron microscopy with energy-dispersive X-ray spectroscopy; µ-Raman—micro-Raman spectroscopy; Raman—Raman spectroscopy; SEM—scanning electron microscopy; FTIR—Fourier transform infrared spectroscopy; ATR-FTIR—attenuated total reflectance–Fourier transform infrared spectroscopy; dw—dry weight.

**Table 3:** Comparison of exposure assessments for MPs in health-related food products.

Product Categories	Type of Product	Country	Estimated Daily Intake (EDI) *	Estimated Annual Intake (EAI) *	Other Exposure Assessment Indices *	Reference
Dietary supplements	Fiber supplements	Australia	children: 0.1–0.48 MPs/day adults: 0.18–4.08 MPs/day	-	-	[24]
	Omega-3 products	Republic of Korea	consumers: 16.3 ± 8.1 MPs/day	-	-	[61]
Herbs and plant-based beverages	Turmeric	Iran	-	613 MPs/person/year	-	[25]
	Red Pepper	Iran	-	544 MPs/person/year	-	[25]
	Black Pepper	Iran	-	188 MPs/person/year	-	[25]
	Tea	Turkey	sage tea: 0.25 MPs/mL/day chamomile tea: 0.34 MPs/mL/day linden tea: 0.34 MPs/mL/day green tea: 0.76 MPs/mL/day	-	-	[51]
Honey		Kosovo	0.37 ± 0.20 MPs/day	136 ± 75 MPs/person/year	-	[26]
		Turkey	industrial honey: 0.16 MPs/day artisanal honey: 0.38 MPs/day	industrial honey: 59.6 MPs/person/year artisanal honey: 138.6 MPs/person/year	-	[86]
		Turkey	1.05 MPs/day	382 MPs/person/year	-	[85]
Infant formulas		Poland	49 ± 32 MPs/day	-	-	[76]
		Poland	58 MPs/day	-		[77]
		Turkey	5.64 MPs/kg bw/day	-	-	[75]
Clinical nutrition products	Enteral nutrition formulas	Turkey	women: 24–61 MPs/day men: 30–76 MPs/day	-	-	[27]
	Parenteral nutrition formulas	Belgium	-	-	lipid emulsion for neonates: 8–115 MPs/72 h crystalloid solution for neonates: 1–52 MPs/72 h	[72]

* Units vary depending on study design; bw—body weight.

## Data Availability

No new data were created or analyzed in this study. Data sharing is not applicable to this article.

## Abbreviations

The following abbreviations are used in this manuscript:

ABS	Poly(acrylonitrile-co-butadiene-co-styrene)
ATR-FTIR	Attenuated total reflectance–Fourier transform infrared spectroscopy
bw	Body weight
dw	Dry weight
EAAC	Ethylene-acrylic acid copolymer
EAI	Estimated Annual Intake
EDI	Estimated Daily Intake
EPDM	Ethylene propylene diene monomer
ES	Etimicin sulfate
EVA	Ethylene-vinyl acetate
FTIR	Fourier transform infrared spectroscopy
GlS	Glucose solution
GS	Grape sugar
GSC	Glucose and sodium chloride
HFFR	Halogen-free flame retardants
HSSC	Hydroxyethyl starch with sodium chloride
ISO	International Organization for Standardization
LDPE	Low-density polyethylene
MCF	MPs Contamination Factor
MH	Moxifloxacin hydrochloride
MPCF	MPs Contamination Factor
MPLi	MPs Pollution Load Index
MPs	Microplastics
PA	Polyamide
PAA	Poly(acrylic acid)
PAN	Polyacrylonitrile
PC	Polycarbonate
PDAP	Poly(diallyl phthalate)
PDME	Predicted MPs daily exposure
PE	Polyethylene
PE-co-PP	Poly(ethylene-co-propylene)
PEPPd	Polyethylene-polypropylene diene
PES	Polyester
PET	Poly(ethylene terephthalate)
PEVA	Poly(ethylene-co-vinyl acetate)
PHI	Polymeric Hazard Index
PLA	Poly(lactic acid)
PMMA	Poly(methyl methacrylate)
POM	Polyoxymethylene
PP	Polypropylene
PS	Polystyrene
PTFE	Poly(tetrafluoroethylene)
PU	Polyurethane
PVAc	Poly(vinyl acetate)
PVC	Poly(vinyl chloride)
PVDC	Poly(vinylidene chloride)
PVF	Poly(vinyl fluoride)
PVP	Polyvinylpyrrolidone
PVS	Poly(vinyl sulfonate)
Py-GC-MS	Pyrolysis-gas chromatography-mass spectrometry
Raman	Raman spectroscopy
ROS	Reactive oxygen species
SAC	Poly(styrene-co-acrylonitrile)
SBR	Sodium bicarbonate ringer
SBS	Styrene-butadiene copolymer
SBSo	Sodium bicarbonate solution
SC	Sodium chloride
SEM	Scanning electron microscopy
SEM/EDX	Scanning electron microscopy with energy-dispersive X-ray spectroscopy
TEM	Transmission electron microscopy
UV	Ultraviolet
WHO	World Health Organization
µ-FTIR	Micro-Fourier transform infrared spectroscopy
µ-Raman	Micro-Raman spectroscopy
